# FANCD2 and DNA Damage

**DOI:** 10.3390/ijms18081804

**Published:** 2017-08-19

**Authors:** Manoj Nepal, Raymond Che, Chi Ma, Jun Zhang, Peiwen Fei

**Affiliations:** 1 Cancer Biology Program, University of Hawaii Cancer Center, Honolulu, HI 96813, USA; Mnepal@cc.hawaii.edu (M.N.); rche@hawaii.edu (R.C.); cma@cc.hawaii.edu (C.M.); 2 Graduate Program of Molecular Biosciences and Bioengineering, University of Hawaii, Honolulu, HI 96813, USA; 3 Department of Laboratory Medicine and Pathology, Mayo Clinic Foundation, Rochester, MN 55905, USA; zhang.jun@mayo.edu

**Keywords:** Fanconi anemia, FANCD2, DNA damage repair, checkpoint, cancer and aging

## Abstract

Investigators have dedicated considerable effort to understanding the molecular basis underlying Fanconi Anemia (FA), a rare human genetic disease featuring an extremely high incidence of cancer and many congenital defects. Among those studies, FA group D2 protein (FANCD2) has emerged as the focal point of FA signaling and plays crucial roles in multiple aspects of cellular life, especially in the cellular responses to DNA damage. Here, we discuss the recent and relevant studies to provide an updated review on the roles of FANCD2 in the DNA damage response.

## 1. Introduction of the Fanconi Anemia Pathway

Fanconi anemia (FA) is a rare human genetic disease displaying various clinical symptoms such as severe bone marrow failure, an extremely high incidence of cancer, and many other congenital defects. The incidence of FA is relatively low, manifesting in less than 1 per 136,000 live births. However, the frequency of normal individuals carrying mutations in any of the FA genes is considerably higher, which is estimated at about 1 in 300. Whereby, the incidence of cancer in FA patients is thought to occur at a rate approximately fifty fold higher than the general population and even several hundred-fold higher for particular malignancies [1]. At the cellular level, FA is characterized by chromosomal abnormalities and hypersensitivity to DNA crosslinking agents, such as mitomycin C (MMC), diepoxybutane (DEB), and Cisplatin [2,3,4]. These common features are displayed in at least 22 known complementation groups FANC-A, B, C, D1, D2, E, F, G, I, J, L, M, N, O, P, Q, R, S, T, U, V and W [5,6,7,8,9,10,11,12,13,14,15,16,17], which suggests that the FA proteins all function in a shared signaling transduction pathway, namely, the FA pathway. This pathway is activated either during DNA replication or upon DNA damage [18,19]. Upon activation, Fanconi anemia group D2 protein (FANCD2) is monoubiquitinated at K561 and works in cooperation with other FA and non-FA proteins [9,11,20,21,22,23,24] to repair DNA damage [5]. In regards to the FA pathway, monoubiquitinated or activated FANCD2 is often portrayed as the functional representative for the functions of activated FA signaling. Within FA signaling or any signaling networks, protein complex formation is essential in regulating signal transduction and FA function. This includes the FA core complex, which is comprised of eight FA proteins and contains the E3 function [6,9,11,21], of which FANCL is the catalytic subunit [25]. Silencing FANCL or any other component of the FA E3 complex is a routine tool used in the laboratory to study the functions of activated versus inactivated FANCD2.

In the past decade, we have been greatly interested in studying the role of the FA signaling pathway in human cancer [26,27]. This pathway appears to be an essential part of the DNA-damage repair-signaling network. Interestingly, four FA-gene products (FANCD1/J/N/S) are previously-known DNA-damage repair proteins (BRCA1/2, BRIP1, and PALB2), which are mainly related to breast cancer susceptibility; and many others (FANCG/O/Q/R/U/V/W) are also previously known proteins involved in DNA damage repair (Rad51, Rad51C, XRCC2/4/9, REV7, and RFWD3) [17,28]. With a considerable number of these functionally-significant players in the FA signaling pathway, it is clear that the FA pathway is greatly important in maintaining genome stability in response to a variety of genotoxic stresses in the general population. Therefore, we believe it is crucial to first investigate the characteristics of FANCD2, the representative of the FA pathway, in order to advance our understanding of how the FA signaling pathway protects human cells from genome instability and thus neoplastic transformation.

## 2. The Importance of Fanconi Anemia Group D2 Protein (FANCD2)

Amongst the FA genes, FANCD2 is the most evolutionarily conserved gene, demonstrating a high sequence similarity from lower eukaryotes to humans [29]. This differs from the other FA genes (with the exception of FANCM [7]), as most of the FA homologs only exist in mammals, acting in concert to respond to DNA damage [30]. DNA crosslinks are one of the few major forms of DNA damage, which presents a major challenge to genomic stability. In eukaryotes, DNA crosslink repair is a complex process; yeast cells require a combination of nucleotide excision repair (NER), homologous recombination (HR) repair, and post replication repair/translesion DNA synthesis (TLS) to remove DNA crosslinks. However, in mammalian cells, the cellular response to DNA crosslinks requires the coordination of complex signaling networks, which includes the FA proteins/pathway [29]. Typically in FA cells, the inability of FANCD2 to be monoubiquitinated appears to be a common molecular defect in response to a variety of genotoxic stresses [12,15,27,31]. Many studies indicate that FANCD2 acts in coordination with many known repair proteins and those yet to be identified, and in nearly all phases of the DNA damage response, damage-sensing, signal transduction, and execution of repair. As such, FANCD2 can perform the signaling transduction role in ATM signaling; specifically, the phosphorylation of FANCD2 at Ser222, initiated by ATM, contributes to arresting cells in the S phase of the cell cycle [32]. Considering checkpoint mechanisms centering on the coordinated events [33], the DNA damage repair function of FANCD2 is equally crucial in arresting or resuming cell proliferation, or in helping eliminate over-damaged cells via apoptosis; however, the latter demands additional studies.

To date, 22 corresponding FA genes have been identified, and their biallelic germline mutations account for the occurrence of FA with the exception of FANCB and FANCR [34,35]. In addition, about 80% of the biallelic germline mutations are attributed to compromised FANCD2 monoubiquitination [36]. As monoubiquitination occurs on the evolutionarily conserved lysine residues, mutations affecting either FANCD2 at K561 or its paralog FANCI at K523 appear to be responsible for major molecular defects, as displayed by the hypersensitivity to DNA damage agents in the FA cells [37,38]. Following monoubiquitination, FANCD2/I is required to work in concert with FANCD1/breast cancer type 2 susceptibility protein (BRCA2), FANCR/RAD51, FANCS/BRCA1, FANCN, FANCJ, FANCO, FANCP, and FANCQ for DNA damage repair [39] in nearly all known repair mechanisms, such as HR, TLS, or postreplication repair, and others [40,41]. To date, accumulated FA studies have indicated that many of the FA and FA-associated proteins not only perform their common role in the FA pathway, but also conduct tasks in an FA pathway-independent manner [42,43,44,45]. Among them, FANCD2 is highly prominent and associated with numerous functions performed independent of the FA pathway. This is further supported by its conservation in regards to its homologous presence in the species, wherein many FA gene-related homologs are absent [27].

## 3. FANCD2 under Stressed Conditions

The cellular stress response is comprised of a network of signaling transduction pathways; when DNA damage occurs, the signaling transduction pathways in this network are well coordinated and divided into phases of sensing, signal transduction, and DNA-lesion repair. Specifically, surveillance proteins can sense DNA damage, initiate cell growth arrest, perform DNA-damage repair, and/or execute cell death programs. These responses inhibit the generation of potentially deleterious cancerous mutations to guard genome stability [46].

### 3.1. Cross Talking with Human Homologs of Yeast Rad6 (HHR6) Signaling

**Figure 1:**
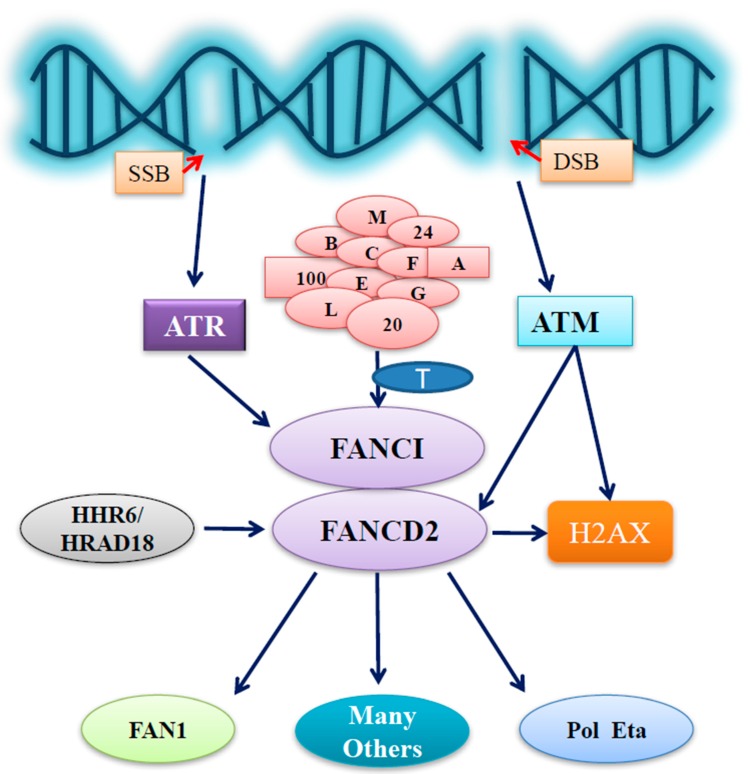
Outline of FA group D2 protein (FANCD2) functions under stressed conditions. In stressed cells, ataxia telangiectasia and Rad3-related protein (ATR) or ataxia telangiectasia mutated (ATM) is activated upon the generation of DNA single or double strand breaks (SSB or DSB), respectively. FANCD2 activation/monoubiquitination issued from the Fanconi anemia (FA) core complex E3 and an E2 (FANCT) can be promoted by the phosphorylation of FANCI triggered by activated ATR, thus conducting important roles that have originated from the activation of ATR. FANCD2 can also play roles not only in aiding ATM signaling for S phase arrest through ATM-dependent phosphorylation at S222 but also possibly in facilitating the initiation of ATM signaling far more upstream via its involvement in the phosphorylation of H2AX. Furthermore, human homologs of yeast Rad6 (HHR6) & hRad18 are also capable of regulating the functions of FANCD2, together influencing the functions of the downstream partners of FANCD2, including Fanconi-associated nuclease 1 (FAN1), DNA polymerase eta (Pol eta), and many others known or yet to be identified.

A similar sensitivity to crosslinking agents is displayed in FA cells as well as in the Rad6^−/−^ yeast-prompted study on the potential link between FANCD2 monoubiquitination and human homologs of yeast Rad6 (HHR6) [47]. Subsequently, HHR6 was found to be capable of regulating FANCD2 monoubiquitination in a distinct manner from FANCT (the ubiquitin conjugating enzyme E2-UBE2T) [47], which cooperates with the FA complex E3 to monoubiquitinate FANCD2 [12]. This observation questioned whether or not hRad18, a functional partner of HHR6, also participated in the regulation of FANCD2 monoubiquitination. To answer this, several studies showed that hRad18 could also regulate FANCD2 monoubiquitination [48,49,50]. Moreover, monoubiquitinated FANCD2 was found to modulate the activity of translesion DNA synthesis [26], at least partly through interacting with pol eta at known regions previously characterized to interact with proliferating cell nuclear antigen (PCNA) [51,52]. Importantly, upon DNA damage, the interaction between pol eta and FANCD2 occurs earlier than that with PCNA [26]. Furthermore, it has been indicated that FANCD2 monoubiquitination performs an anchoring role, similar to the histone proteins to bind DNA in general, but more specific in regards to FANCD2 regulation of pol eta relocation at the site of damaged DNA [26]. Additionally, FANCD2 monoubiquitination can also occur in vitro in the absence of the FA core complex E3 [53], evidently showing it can act in an FA pathway-independent manner. Together, these studies indicate that in the early phases of the DNA damage response, FANCD2 plays a crucial role as a sensor as well as a messenger for the timely repair of damaged DNA (Figure 1).

### 3.2. Coupling with Ataxia Telangiectasia and Rad3-Related Protein (ATR)/Ataxia Telangiectasia Mutated (ATM) Signaling

In response to genotoxic stresses, the FA pathway activates the FA core complex harboring the activity of E3 ubiquitin ligase, which in turn leads to the monoubiquitination of FANCI and FANCD2 (Figure 1). The monoubiquitinated FANCI-FANCD2 complex is recruited to DNA damage sites and promotes TLS, NER, and Rad51-medated HR [54,55]. Ataxia telangiectasia mutated (ATM) along with its regulator, the MRN (Mre1 1-Rad50-NBS1) complex, sense double strand breaks (DSBs) [56]. Whereas, ATR with its regulator ATRIP (ATR-interacting protein) sense single strand DNA (ssDNA) that was generated by processing DSBs, as well as the ssDNA present at the stalled replication forks. Both kinases then phosphorylate proteins to initiate signaling cascades, which includes checkpoint kinases (CHK1) and (CHK2), both of which can initiate a secondary wave of phosphorylation events to extend signaling and promote DNA-damage repair signaling [57]. Typically, ionizing radiation can lead to ATM phosphorylation of FANCD2 and Nijmegen breakage syndrome protein 1 (NBS1), and an S-phase arrest [58]. Thus, FANCD2 not only performs a critical role in orchestrating the FA proteins in the FA signaling pathway but also closely cooperates with ATM to issue an S phase arrest to modulate cell proliferation and eventually prevent cells from genome instability.

In the FA pathway, the FANCD2/I complex has been known for its close partnership between FANCD2 and its paralog FANCI as early as 2007 when FANCI was identified [59]. Studies have shown that many functions of FANCD2 in the FA signaling pathway are largely dependent on the phosphorylation of FANCI that is performed by ATR [60]. This is consistent with the non-ubiquitinated state of the FANCD2-FANCI complex when recruited to DNA interstrand crosslinks [61]. BLM was also found to be involved in the activation of FANCD2 in stressed cells [62]. Certainly, this discovery promotes its function in maintaining genome stability, which includes a newly identified role in governing the stability of replication forks [63]. FANCD2 was also found to be required for proper phosphorylation of H2AX (a variant of the H2A protein family) and hence activation of ATM in rhabdomyosarcoma Rh30 cells, but not essential for ATR-Chk1 activation. This observation spans beyond the roles that FANCD2 plays in response to ICL damage [54,55] (as the focal point in the FA signaling pathway). Here, FANCD2 acts more like a sensor, similar to those sensors passing signaling in the early phase of the DNA damage response (DDR). This substantially contributes to ATM functions in maintaining genome integrity in response to DNA DSB [64]. Furthermore, FANCD2 was also found to be capable of modulating the enzymatic activities of FAN1 and pol eta [26,65,66,67,68], needed in the later phase of the DDR for DNA damage repair. However, whether FANCD2 directly plays some enzymatic roles in the DNA damage responses waits to be further investigated, although its nuclease activity was proposed [69].

### 3.3. Cooperating with Other Signaling Pathways

Previously, studies have suggested that signaling pathways such as PI3K and Ras are deregulated during the progression of human malignancy. Additionally, the defective FA pathway function is also a likely contributor during the course of the neoplastic transformation, which originates from these defective signaling pathways. Moreover, in yeast, mechanistic target of rapamycin (mTOR) promotes cell survival but at the cost of an increase in the alkylation agent melphalan, accompanied with significant down-regulation of FANCD2 [70]. Experiments have demonstrated that mTOR signaling controls FANCD2 gene transcription via cyclin-dependent kinase 4 (CDK4), supporting the observation that FANCD2 is regulated by Rb-E2F1(retinoblastoma protein-E2 transcription factor 1) [71]. Furthermore, Ras, p53, and Rb signaling was also found to be able to circuit the signaling control of FANCD2 by CDK4 [72]. This suggests that cancer cells with self-sufficiency in growth signaling and resistance to anti-proliferation signaling may depend on the functional status of FANCD2 for survival and the activation of the DNA damage checkpoint mechanisms discussed above. However, this field of studies regarding the FA proteins is relatively under-investigated, requiring more studies to continue.

## 4. FANCD2 in Non-Stressed Condition

As viewed above, huge attention has been given to the stress condition of cells where monoubiquitination/activation of FANCD2 takes place to eventually repair damaged DNA. In contrast, little attention has been given to FANCD2 under normal (non-stressed) conditions. In non-stressed cells, the FA pathway is not constitutively active; rather, it is activated in the S phase of the cell cycle [31]. This basal level of FANCD2 monoubiquitination occurring in normally growing cells has been demonstrated to be essential for replication origins to fire at a normal rate [73]. Conversely, the loss of the basal level of FANCD2 monoubiquitination leads to a slow rate of replication origin firing. For the first time, this observation was able to give a rational explanation of the aging phenotype displayed in many FA patients. With the studies focused on the FA proteins for their roles in resolving stalled replication forks [62,74], whether FANCD2 also plays roles in replication elongation and/or termination appears to be another important aspect for future research on FANCD2 under non-stressed conditions. Recently, with the progress of many modern research technologies, metabolomics has attracted many cancer researchers to study the cancer mechanism at the metabolic level. Accordingly, the first metabolomics study for the tumor promotion role played by a compromised FA signaling pathway filled the bank for this related field of studies in metabolism, consistent with the well-accepted concept that cancer is one of four major metabolic disorders, which also include aging, diabetes, and stroke [75]. These recent studies on the involvement of FANCD2 in metabolism via the mitochondria [36,76] further indicate the importance of FANCD2 functions in non-stressed cells, which unveil crucial biological functions that were not previously considered.

## 5. Conclusive Remarks

FANCD2 critically governs the FA signaling pathway by interacting with FA and non-FA protein partners for DNA damage repair to guard genome stability. On the other hand, FANCD2 can also function as a veteran checkpoint-player (Figure 1), coupling with a variety of cellular processes outside the FA signaling pathway. As DNA damage causes cancer, it is also acknowledged that, conversely, DNA damage is also beneficial to the efficacy of cancer treatments. Recently, the development of molecular inhibitors has become a promising therapeutic strategy to target DNA-damage repair by inhibiting the DNA repair process. From viewing functions focused on FANCD2, the inhibition of a DNA-damage repair player or even a whole process is harder to achieve than expected, because the redundancy, overlapping, and multifaceted natures are rooted in the human DNA repair signaling network, for which we shall need to have more in-depth studies to get into the bottom of these properties.
